# Cultural influences on social feedback processing of character traits

**DOI:** 10.3389/fnhum.2014.00192

**Published:** 2014-04-04

**Authors:** Christoph W. Korn, Yan Fan, Kai Zhang, Chenbo Wang, Shihui Han, Hauke R. Heekeren

**Affiliations:** ^1^Department of Education and Psychology, Freie Universität BerlinBerlin, Germany; ^2^Berlin School of Mind and Brain, Humboldt Universität zu BerlinBerlin, Germany; ^3^Dahlem Institute for Neuroimaging of Emotion, Freie Universität BerlinBerlin, Germany; ^4^Psychiatric Hospital, University of ZurichZurich, Switzerland; ^5^Cluster of Excellence “Languages of Emotion,” Freie Universität BerlinBerlin, Germany; ^6^Department of Psychiatry, Charité-Universitätsmedizin Berlin, Campus Benjamin FranklinBerlin, Germany; ^7^Department of Psychology, Peking UniversityBeijing, China

**Keywords:** interdependence, independence, social conformity, positivity biases, medial prefrontal cortex

## Abstract

Cultural differences are generally explained by how people see themselves in relation to social interaction partners. While Western culture emphasizes independence, East Asian culture emphasizes interdependence. Despite this focus on social interactions, it remains elusive how people from different cultures process feedback on their own (and on others') character traits. Here, participants of either German or Chinese origin engaged in a face-to-face interaction. Consequently, they updated their self- and other-ratings of 80 character traits (e.g., polite, pedantic) after receiving feedback from their interaction partners. To exclude potential confounds, we obtained data from German and Chinese participants in Berlin [functional magnetic resonance imaging (fMRI)] and in Beijing (behavior). We tested cultural influences on social conformity, positivity biases, and self-related neural activity. First, Chinese conformed more to social feedback than Germans (i.e., Chinese updated their trait ratings more). Second, regardless of culture, participants processed self- and other-related feedback in a positively biased way (i.e., they updated more toward desirable than toward undesirable feedback). Third, changes in self-related medial prefrontal cortex activity were greater in Germans than in Chinese during feedback processing. By investigating conformity, positivity biases, and self-related activity in relation to feedback obtained in a real-life interaction, we provide an essential step toward a unifying framework for understanding the diversity of human culture.

## Introduction

Culture shapes various aspects of human cognition (Nisbett et al., 2001; Nisbett and Masuda, 2003; Henrich et al., 2010; Heine, 2012). A prominent framework that integrates diverse cultural differences centers on how people relate to those with whom they interact (Markus and Kitayama, 1991, 2010; Singelis, 1994; Oyserman et al., 2002; Triandis and Suh, 2002; Heine and Buchtel, 2009): members of independent (or individualistic) cultures (e.g., Western cultures) construe their selves as distinct from others whereas members of interdependent (or collectivistic) cultures (e.g., East Asian cultures) construe their selves as interconnected with close others. Yet, how culture influences the processing of social feedback from others has not been investigated—which is surprising given that the relation between self and others defines self-construal. Here, we compared how members of an independent culture (Germans) and from an interdependent culture (Chinese) process social feedback on character traits. Social feedback processing comprises several components and mechanisms pertinent for social cognition in general. Specifically, when people receive social feedback they can take it into account to different degrees. That is, they can show various amounts of social conformity to social feedback in general and they can show differential processing of desirable and undesirable social feedback—both of which may differ across cultures. Our study aimed at providing a broad picture of possible cultural influences on social feedback processing and we thus investigated its behavioral and neural aspects.

Social feedback processing comprises social conformity because people often conform their own views to the social feedback provided by others (Bond and Smith, 1996). In independent cultures, the concept of conformity bears a rather negative connotation whereas in interdependent cultures it bears a rather positive connotation (Kim and Markus, 1999). Meta-analytic evidence indicates that members of interdependent cultures conform more to social information in classic Asch-type line judgment tasks (Bond and Smith, 1996). However, the authors of the meta-analysis concede that line judgment tasks limit the concept of conformity to cases where participants can only conform—or not—to objectively incorrect statements about unambiguous physical stimuli (i.e., the lengths of two lines). Conforming to feedback on character traits (i.e., to information that is open to interpretation) differs from conforming to statements about physical stimuli, and directly relates to the concept of self-construal.

Social feedback can be processed differently depending on whether the feedback is desirable or undesirable (Korn et al., 2012). This component of social feedback processing can be closely related to the literature on positivity biases (for overviews see Taylor and Brown, 1988; Heine and Hamamura, 2007; Leary, 2007). We have previously shown that Germans engage in positively biased social feedback processing. That is they take desirable vs. undesirable self- and other-related feedback more into account (Korn et al., 2012). This finding fits well with a large literature indicating that Westerners show positivity biases (Taylor and Brown, 1988). However, it is debated whether East Asians are also prone to positivity biases and in particular whether they show self-enhancement (Sedikides et al., 2003, 2007) or not (Heine and Hamamura, 2007; Heine et al., 2007). One important example of a bias in social perception that differs across cultures is the self-advantage in face recognition. Both Westerners and East Asians respond faster to pictures of their own faces than to pictures of familiar faces and this effect can be reduced via self-threat, which indicates that self-positivity mediates the effect (Ma and Han, 2010). Interestingly, East Asians show a smaller self-advantage (Sui et al., 2009). Thus, previous studies investigating positivity biases in the perceptual domain point to cultural differences. Here, we aimed at adding to this debate by using feedback processing as a novel approach to assess possible cultural differences in positivity biases.

On the neural level social feedback processing is supposed to be mediated by regions that play a role in self- and other-related processes. Self- and other-related neural activity has been the central focus of cultural neuroscience (Han and Northoff, 2008; Vogeley and Roepstorff, 2009; Kitayama and Uskul, 2011; Han et al., 2013). Differences in interdependent and independent self-construal have been linked to the anterior cingulate cortex and the medial prefrontal cortex (ACC/MPFC) (Zhu et al., 2007; Chiao et al., 2009a,b; Ng et al., 2010; Ma et al., 2014), which play an important role in various aspects of social cognition such as judging character traits (Heatherton, 2011; Denny et al., 2012; Wagner et al., 2012) and engaging in mentalizing or theory-of-mind (i.e., inferring the mental states of others) (Mar, 2011; Frith and Frith, 2012). For example, MPFC activity was higher in Westerners compared with East Asians when they judged whether character traits, social roles or physical attributes were self-descriptive (Ma et al., 2014). However, these previous studies have not addressed how MPFC activity is modulated by self- or other-related information.

In the current study, we approached social feedback processing as a whole and therefore tested hypotheses on the behavioral and neural level. Specifically, we tested for cultural influences on social conformity, positivity biases, and ACC/MPFC activity by investigating how German and Chinese participants process character trait information obtained within the context of a real-life social interaction. First, we hypothesized that Chinese conform more to social feedback on character traits than Germans. Second, we investigated whether members of both cultural groups differed in positively biased feedback processing. Third, we expected cultural differences in ACC/MPFC activity when participants received social feedback.

Additionally, we explored cultural differences in activity for reward- and comparison-related components of social feedback, which we described in our previous study (Korn et al., 2012). The reward-related component is operationalized as a positive correlation with the “positivity” of social feedback for self (e.g., receiving the feedback “you are extremely helpful” is conceptualized as more rewarding than receiving the feedback “you are somewhat helpful;” see also Izuma et al., 2008). The reward-related component is associated with activity in the ventral striatum and in the ACC/MPFC (Izuma et al., 2008; Korn et al., 2012). The comparison-related component captures that people commonly have their own view before they receive social feedback (e.g., they may think that they are “quite friendly” but then receive the social feedback that they are “very friendly” or that they are “not friendly”). Thus, the comparison-related component operationalizes the discrepancies between people's pre-existing views and the feedback they receive. This component is associated with activity in the mentalizing network (Korn et al., 2012).

In line with common practice in research on cultural comparisons we used nationality as a proxy for cultural group membership but additionally assessed participants' explicit endorsement of independence and interdependence (Singelis, 1994; Henrich et al., 2010; de Greck et al., 2012; Ma et al., 2014). Living in a foreign culture could increase conformity due to possible stress and insecurity (Sam and Berry, 2010; Heine, 2012) or in-group/out-group effects (Bond and Smith, 1996). It can also be expected that individuals who move abroad may be more independent (and less interdependent) in general (Kitayama et al., 2006, in press) or that they may be more similar to their host culture. These potential confounds are thus especially pertinent in relation to our first behavioral hypothesis about overall cultural differences in social conformity. To minimize these potential confounds, we obtained behavioral data from both German and Chinese participants in both Berlin and Beijing (Table 1). In Berlin, we collected functional magnetic resonance imaging (fMRI) data to test for cultural differences in ACC/MPFC activity. If living abroad entails neural differences, these should be mediated by differences in behavior (e.g., differences in independence, interdependence and/or social conformity). We therefore collected behavioral measures in both countries but fMRI data only in one country.

**Table 1:** **Characteristics of participants (data are given as mean and standard deviation)**.

	**Germans**		**Chinese**	
	**Berlin fMRI**	**Beijing behavior**	**Berlin fMRI**	**Beijing behavior**
*n*	27	24	28	25
Sex, female	14	10	14	15
Age, years (y)	24.3 (2.47)	24.3 (3.24)	25.9 (2.53)	22.7 (1.86)
Education, y	16.1 (2.22)	16.9 (1.77)	18.5 (3.13)	16.1 (2.39)
Living without parents, y	4.6 (2.84)	4.6 (3.49)	6.5 (6.08)	5.8 (4.19)
Living abroad, y	–	0.9 (0.91)	0.8 (0.45)	–
Learning foreign language, y	–	2.0 (1.72)	1.7 (2.08)	–
Interdependence score	3.10 (0.53)	3.58 (0.38)	3.71 (0.38)	3.89 (0.41)
Independence score	3.72 (0.30)	3.85 (0.32)	3.58 (0.36)	3.47 (0.43)
Self-esteem score	23.0 (5.35)	23.6 (3.57)	20.6 (5.40)	21.7 (4.36)
Perceived similarity score	3.67 (1.47)	4.06 (1.63)	3.79 (1.77)	4.24 (1.13)

## Methods

### Participants

We recruited participants of German and Chinese cultural origin in Berlin and Beijing via flyers, word-of-mouth, and mailing lists (e.g., by the German Academic Exchange Service; German Berlin: *n* = 27, German Beijing: *n* = 24, Chinese Berlin: *n* = 28, Chinese Beijing: *n* = 25) (Table 1). Our study employed a 2 (culture: German/Chinese) by 2 (place: Berlin/Beijing) between-subject design. The two groups in Berlin underwent fMRI scanning while the two groups in Beijing were tested behaviorally. Data from the German fMRI group have been reported previously (Korn et al., 2012). Participants were excluded if they showed excessive head movement (> 6 mm and/or > 3°). In the German group in Berlin three of the initial 30 participants had to be excluded (one showed excessive head movement, another did not tolerate the scanner environment, and data from another subject could not be used due to technical problems with the script). In the Chinese group in Berlin two of the initial 30 participants had to be excluded because of excessive head movement (one of these two additionally reported not understanding the task at the end of the experiment). All scanned participants were right-handed.

All German participants spoke German as their mother-tongue. All Chinese participants were fluent in Mandarin and spoke Mandarin or Cantonese as their mother-tongue. All except three German participants had been raised by two German parents; three participants had one German parent and one English, French, or Russian parent. All Chinese participants had been raised by two Chinese parents. In the Chinese group in Berlin four participants were from Hong Kong. German and Chinese participants were recruited, instructed, and tested in German and Mandarin, respectively. All participants gave written informed consent.

### Experiment

#### Overview

**Figure 1:**
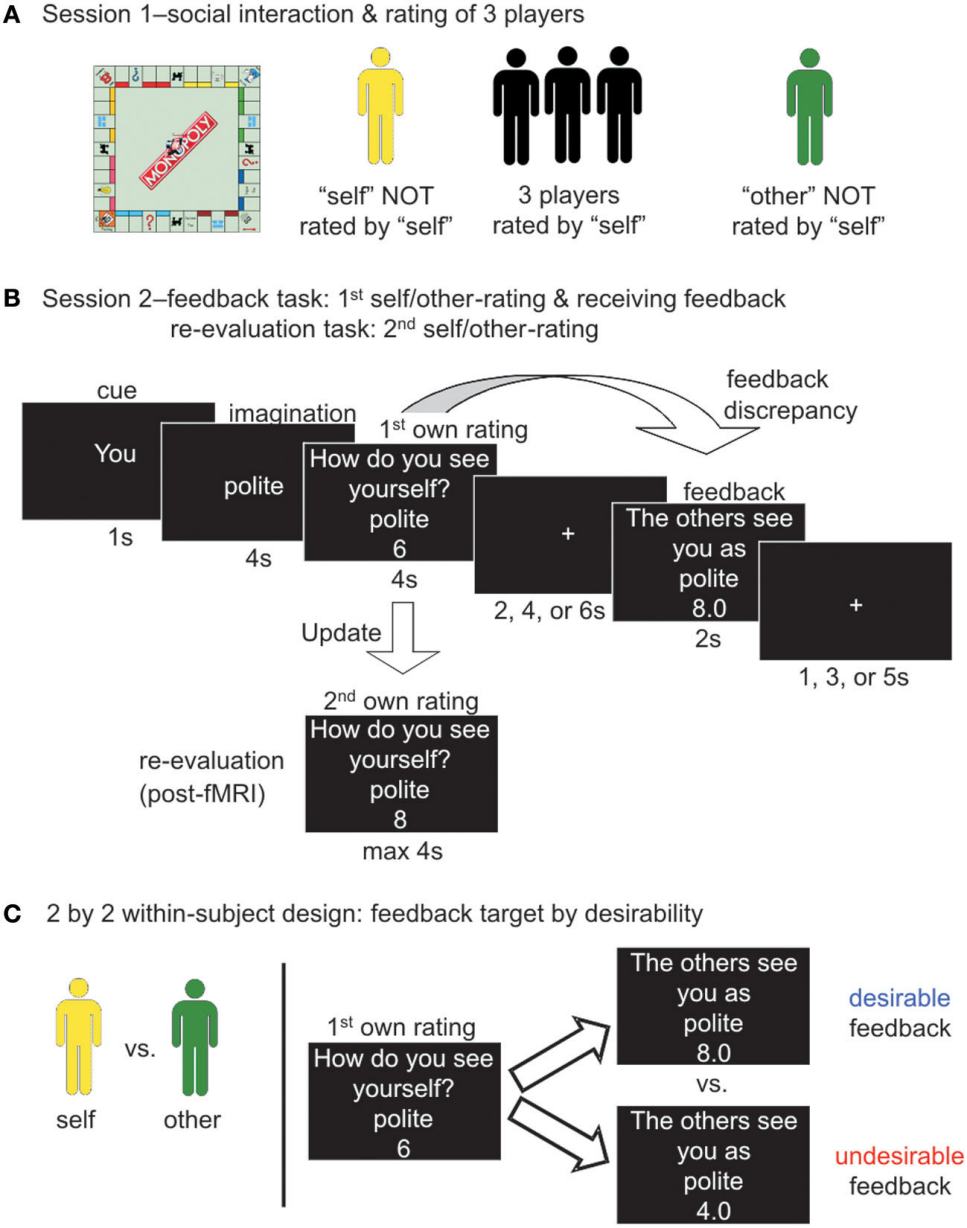
**Experimental design—receiving social feedback from peers after a real-life interaction. (A)** Participants came to the laboratory in groups of either five German or five Chinese participants. In the first session of the experiment, participants got to know each other by playing the board game “monopoly” for 1 h 15 min. Afterwards, each person rated three of the other players on 40 positive and 40 negative trait adjectives (Supplementary Table S1) on a Likert scale from 1 (this trait does not apply to the person at all) to 8 (this trait applies to the person very much). Participants did not rate themselves (yellow) and did not rate one of the other players (green). **(B)** In the second session, participants in Berlin performed the feedback task in the fMRI scanner and participants in Beijing performed the feedback task on a PC. In each trial, participants first saw a cue indicating whether the trial was about themselves or about the other person whom they had not rated during the first session. They had to imagine how much one of the 80 traits applied to themselves or to the other person. They first gave their own rating and then saw the feedback rating in form of the mean rating they believed three other participants had given during the first session. The absolute differences between participants' own ratings and the feedback ratings they received was conceptualized as feedback discrepancies and manipulated. Afterwards, all participants performed the re-evaluation task behaviorally on a PC. Participants rated themselves and the other player a second time so that we could assess how much they updated their ratings. **(C)** For the main behavioral analyses we employed a design with four factors. There were two within-subject factors (depicted here): feedback target (self/other) and feedback desirability (desirable/undesirable). Feedback was desirable feedback when feedback ratings were higher than participants' own first ratings and undesirable when feedback ratings were lower than participants' first ratings. All ratings for negative trait adjectives were reverse-coded. Thus, feedback desirability was independent of the valence of the trait adjective. The two between-subject factors were culture (German/Chinese) and current place of residence (Berlin/Beijing; Table 1).

The experimental procedure has been adapted from our previous study (Korn et al., 2012) (Figure 1) and consisted of two sessions. We wanted participants to believe that they would get realistic feedback from peers of the same culture with whom they had interacted in real-life. During the first session (Figure 1A) each participant interacted with four other participants for 1 h and 15 min by playing a popular board game. Consequently, each participant rated three of the four other participants on 40 positive and 40 negative trait adjectives. See Stimuli and translation, Social interaction and rating of 3 players (first session), and Supplementary Table S1. In the second session, participants believed that they would receive the mean rating of three other participants on each adjective as feedback. See Figure 1B and Feedback task and re-evaluation task (second session). We tested how much participants took this feedback into account by asking them to rate their own personality before and after receiving social feedback (i.e., in the feedback and in the re-evaluation tasks). Additionally, each participant rated one other person before and after receiving social feedback for this person. Since participants in Berlin underwent fMRI scanning while receiving feedback, they performed the two sessions on two consecutive days. Participants in Beijing performed the two sessions on the same day.

#### Stimuli and translation

We used 40 positive and 40 negative trait adjectives as described previously (Korn et al., 2012). See Supplementary Table S1 for a list of trait adjectives. Trait adjectives were translated from German into Mandarin by an accredited court interpreter and double-checked by a native Mandarin speaker. One German and two Chinese authors made sure that German and Mandarin versions of the trait adjectives captured the same meaning. All instructions were translated by two Chinese authors.

To confirm that participants perceived the trait words as positive and negative in the way we had predefined them, participants rated all 80 trait adjectives on social positivity on a Likert scale from 1 (not positive at all) to 8 (very positive) at the very end of the experiment. Five participants in the German Beijing group did not complete this desirability rating due to time constraints. Across all participants, mean ratings for positive and negative trait words differed from the midpoint of the scale as assessed by one-sample *t*-tests [mean rating: positive words = 6.66, *SD* = 0.58; *t*_(98)_ = 36.68, *p* < 0.001; negative words = 2.63, *SD* = 0.66; negative words *t*_(98)_ = −28.12, *p* < 0.001]. Positive trait adjectives were rated similarly by both cultural groups but negative trait adjectives were rated as less desirable by German compared with Chinese participants [independent sample *t*-test: *t*_(97)_ = −2.86, *p* = 0.005]. However, this difference did not compromise our findings related to updating behavior since the factor valence did not interact with any other factors (see Additional behavioral results).

#### Social interaction and rating of 3 players (first session)

The first session aimed at creating a real-life interaction among peers so that the social feedback would be meaningful for participants. For the first session of the experiment (Figure 1A), participants came into the laboratory in groups of five people of the same culture and got to know each other by playing a table-top version of the popular board game “Monopoly” (HASBRO, Soest, Germany; HASBRO, Shanghai, China) for 1 h and 15 min. We made sure that participants did not know each other before the experiment.

In the groups in Berlin and in the Chinese groups in Beijing all five participants in a group were of the same sex. German groups in Beijing consisted of members of both sexes since we were unable to recruit enough German participants in Beijing to form same sex groups. Additionally, one of the German participants in Beijing was aware of the experimental manipulation and only participated so that we could form a group of five people. Data from this participant were not analyzed. Therefore, the total number of German participants in Beijing was 24 and not 25.

We chose the board game “Monopoly” for the social interaction because it is highly engaging, quite well-known, and allows players to show a variety of cooperative and competitive behaviors. Furthermore, within 1 h 15 min nobody was eliminated from the game. The rules of the game were explained to all participants before the game. The study was introduced as a study about how people get to know each other. Specifically, we explained participants that the study mirrored situations such as meeting new people at the first day of joining a club or starting a new job. In many social situations, people are motivated to form a picture of other persons' personality and at least implicitly take into account that in turn others may judge their personality and give social feedback. We wanted to exclude that in our study some but not all participants expected that they would have to rate the others and receive feedback. Therefore, participants were told before they started to play the game that they were going to be rated by the other players of their group and they believed that their own ratings were going to be shown to the other players in an anonymous fashion. During the game participants were free to talk about whatever topics they wanted. Participants wore name tags and we made sure that participants knew the names of all players after the game. After 1 h 15 min we assessed the ranking of the participants in the game, i.e., assigned the first rank to the winner and so on. Participants' ranks in the game did not correlate with any behavioral measures on the task as assessed by Spearman correlations (all *p* > 0.1).

After the game, each participant rated three of the four other participants on 80 trait adjectives on a Likert scale from 1 (this trait does not apply the person at all) to 8 (this trait does apply the person very much); for trait adjectives see Stimuli and translation and Supplementary Table S1. Ratings were given on a PC using the MATLAB toolbox Cogent 2000. Each of the three persons was rated in a separate block. On each trial participants saw one of the 80 adjectives with the first name of the person to rate and had up to 10 s to respond. At the end of the first session of the experiment each participant had rated three other participants and in turn each participant had been rated by three other participants. Participants had not yet rated themselves (depicted in yellow in Figure 1A) and had not yet rated one other player (depicted in green).

#### Feedback task and re-evaluation task (second session)

In the second session of the experiment (Figure 1B), participants performed the following feedback task, either in the MRI scanner (Berlin groups) or behaviorally on a PC (Beijing groups). The feedback task was presented using the MATLAB toolbox Cogent 2000 (www.vislab.ucl.ac.uk/cogent.php). On each trial, participants first saw a cue (1 s) indicating whether the trial was about themselves (“you”) or about the fourth other participant whom they had not rated during the first main part of the experiment (name of the other person). Then, they saw one of the 80 trait adjectives and had to think about how much that trait applied to themselves or to the other person (imagination phase, 4 s). Afterwards, participants were prompted to indicate their rating on an 8-point Likert scale via two button boxes with four buttons each (rating phase, 6 s). After a jittered fixation cross (2, 4, or 6 s) participants saw what they believed to be the mean rating of three other participants from the first session of the experiment (feedback phase, 2 s). This mean rating, which served as the feedback rating, was a number with one decimal, ranging from 1.0 to 8.0 in steps of 0.3. The feedback rating was determined by the program during the experiment to reliably create a sufficient number of trials in which participants received desirable and undesirable feedback (see Task conditions and behavioral analyses). After a second jittered fixation cross (1, 3, or 5 s) a new trial began. Participants performed 4 training trials. The feedback task was split up into four runs. In each of the 4 runs, 20 different (out of the total 80) adjectives were used. Within a single run, participants saw the 20 trait adjectives (10 positive and 10 negative) twice, one time in the self-condition and one time in the other condition. Trials for self and other were randomly intermixed. Adjectives were randomly assigned to the four blocks for each person.

After the feedback task, all participants performed the re-evaluation task outside the MRI scanner on a PC using the MATLAB toolbox Cogent 2000. Participants gave a second rating so that we could measure how much they changed their self- and other-ratings after having received social feedback in the feedback task. Specifically, they rated themselves and the other person again on all 80 trait adjectives in two separate blocks (one for themselves and one for the other person). These blocks were randomized for order. For each trait adjective, participants had up to 6 s to respond.

#### Task conditions and behavioral analyses

The main behavioral analyses employed a 2 by 2 by 2 by 2 design with the within-subject factors feedback target (self/other) and feedback desirability (desirable/undesirable) (Figure 1C) as well as the between-subject factors culture (German/Chinese) and place (Berlin/Beijing) (Table 1). For each participant, trials were classified according to whether feedback was desirable or undesirable. For a positive trait adjective, desirable feedback indicated that the feedback rating was numerically higher than the initial rating (e.g., a participant's initial rating for “polite” was 6 and the feedback rating was 8). For a negative trait, desirable feedback indicated that the original feedback rating was numerically lower than the original initial rating (e.g., a participant's initial rating for “aggressive” was 3 and the feedback rating was 1). Conversely, undesirable feedback was defined as feedback ratings that were more “negative” than participants' own initial ratings (e.g., initial rating of 6 and feedback rating of 4 for “polite” or initial rating of 3 and feedback rating of 5 for “aggressive”). Thus, feedback desirability was independent of the valence of the trait word and we reverse-coded ratings for negative trait adjectives. For each trial (i.e., for each trait adjective; separately for self- and other-conditions) we calculated a “feedback discrepancy” term as the absolute difference between first own ratings and feedback ratings. (Trials with adjectives for which participants failed to respond in time for the first or second rating were excluded.)

(1) $$\begin{array}{l} \text{Feedback discrepancy}=\text{abs}(\text{feedback rating}\\ \text{ —first own rating}) \end{array}$$

This feedback discrepancy term indicated the social comparison-related component of receiving social feedback. Since feedback discrepancies were an independent variable of our task we manipulated their magnitude using a random number generator (see Korn et al., 2012 for details). Trials with a feedback discrepancy of zero were excluded from behavioral analyses since these trials could not be clearly assigned to either receiving desirable or receiving undesirable feedback (see Table 2 for final numbers of trials). To assess how much participants changed their self-concept after receiving social feedback, we calculated an update term quantifying how much participants changed their own ratings.

(2) $$\begin{array}{l} \text{Update}=\text{second own rating}\\ \text{ —first own rating} \end{array}$$

**Table 2:** **Task-related variables**.

	**Germans**				**Chinese**			
	**Berlin fMRI**		**Beijing behavior**		**Berlin fMRI**		**Beijing behavior**	
	**self**	**other**	**self**	**other**	**self**	**other**	**self**	**other**
*n* trials final^a^	72.8 (3.10)	71.3 (2.70)	68.7 (6.37)	66.3 (7.50)	69.5 (3.96)	69.3 (5.61)	70.2 (5.78)	69.2 (6.11)
*n* trials excluded: missing answers	1.70 (1.92)	2.22 (1.97)	4.54 (3.40)	6.50 (5.33)	3.64 (2.63)	3.89 (4.00)	3.00 (2.65)	2.80 (3.16)
*n* trials excluded: zero feedback discrepancies	5.52 (2.29)	6.44 (2.50)	5.08 (2.06)	5.58 (1.93)	6.18 (2.68)	6.14 (1.99)	5.24 (2.07)	6.40 (3.00)
First ratings	5.63 (0.61)	5.22 (0.69)	5.64 (0.57)	5.41 (0.82)	5.60 (0.92)	5.52 (0.65)	5.54 (0.55)	5.42 (0.57)
Second ratings	5.74 (0.61)	5.36 (0.76)	5.84 (0.59)	5.60 (0.87)	5.76 (0.93)	5.64 (0.59)	5.82 (0.63)	5.65 (0.58)
Relative absolute mean update: desirable	0.29 (0.23)	0.31 (0.20)	0.40 (0.26)	0.40 (0.23)	0.41 (0.31)	0.41 (0.27)	0.47 (0.23)	0.48 (0.21)
Relative absolute mean update: undesirable	0.08 (0.15)	0.14 (0.24)	0.07 (0.20)	0.13 (0.22)	0.11 (0.16)	0.15 (0.19)	0.06 (0.22)	0.11 (0.19)
Absolute memory error: desirable^b^	1.35 (0.34)	1.46 (0.28)	1.43 (0.33)	1.51 (0.29)	1.35 (0.42)	1.51 (0.48)	1.30 (0.34)	1.51 (0.39)
Absolute memory error: undesirable^b^	1.18 (0.26)	1.43 (0.24)	1.41 (0.42)	1.52 (0.26)	1.32 (0.32)	1.54 (0.31)	1.21 (0.25)	1.45 (0.30)

a Two participants in the German Beijing group, one participant in the Chinese Berlin and two participants in the Chinese Beijing group completed only three out of four feedback runs due to technical problems.

b Three participants in the German Beijing group did not complete the memory test and five did not complete the desirability rating of the stimuli due to time constraints.

We expected participants to change their ratings on average toward the feedback ratings. That is, for desirable feedback (i.e., feedback ratings higher than own first rating) participants should increase their ratings (i.e., positive updates). For undesirable feedback (i.e., feedback ratings lower than own first rating) participants should decrease their ratings (i.e., negative updates). To test for differences in updating, we first calculated relative mean updates for each participant within each condition by dividing mean updates by the respective mean feedback discrepancies. We then took the absolute value of relative mean updates (i.e., the negative sign of updates following undesirable feedback is changed).

(3) $$\begin{array}{l} \text{Relative absolute mean update}=\text{absolute mean update}\\ \text{ /mean feedback discrepancy} \end{array}$$

Relative absolute updates can be interpreted in a straightforward way; e.g., a relative update of 0.3 indicates that the change in ratings was on average 30% of the difference between initial own ratings and feedback ratings. Overall group differences in relative absolute mean updates indicate group differences in social conformity. Larger relative absolute mean updates for desirable vs. desirable feedback indicate positively biased updating.

#### Memory task

After rating themselves and the other person a second time (i.e., after the re-evaluation task) (Figure 1B), participants performed a memory task on a PC using the MATLAB toolbox Cogent 2000. For all 80 trait adjectives participants had to recollect the feedback they had seen in the feedback task and had to type in that number, i.e., a number between 1 and 8 with one decimal such as 1.0, 1.3, or 1.7. Participants had to recollect the feedback in two separate blocks (one for themselves and one for the other person), which were randomized for order. They had up to 12 s to respond. Three participants in the German Beijing group did not complete the memory test due to time constraints.

Memory errors were calculated as the absolute differences between the recollected number and the actual feedback rating.

(4) $$\begin{array}{l} \text{Absolute memory error}=\text{abs}(\text{feedback rating}\\ \text{ —recollection of feedback rating}) \end{array}$$

Similar to update scores, mean absolute memory errors were compared in a 2 (target: self/other) by 2 (desirability: desirable/undesirable) by 2 (culture: German/Chinese) by 2 (place: Berlin/Beijing) repeated measures ANOVA.

#### Individual difference scores

Participants completed the 24-item version of the Singelis self-construal scale (Singelis, 1994) and the Rosenberg self-esteem scale (Rosenberg, 1965). They rated how similar they perceived the other person on a Likert scale from 1 (not similar at all) to 8 (very similar).

### fMRI data

#### Acquisition

fMRI data were acquired on a 3T scanner (Trio, Siemens, Erlangen, Germany) using a 12-channel head coil. Functional images were acquired with a gradient echo T2^*^-weighted echo-planar sequence (*TR* = 2000 ms, *TE* = 30 ms, flip angle = 70, 64 × 64 matrix, field of view = 192 mm, voxel size = 3 × 3 × 3 mm^3^). A total of 37 axial slices (3 mm thick, no gap,) were sampled for whole brain coverage. Imaging data were acquired in four separate 349-volume runs of 11 min 38 s each. The first five volumes of each run were discarded to allow for T1 equilibration. A high-resolution T1-weighted anatomical scan of the whole brain was acquired (256 × 256 matrix, voxel size = 1 × 1 × 1 mm^3^).

#### Preprocessing

fMRI data were preprocessed using standard procedures in SPM8 (www.fil.ion.ucl.ac.uk/spm). EPI images were realigned, unwarped, co-registered to the respective participant's T1 scan, normalized to a standard T1 template based on the Montreal Neurological Institute (MNI) reference brain, resampled to 3 mm isotropic voxels, and spatially smoothed with an isotropic 8 mm full width at half maximum (FWHM) Gaussian kernel. Using the East Asian brain template provided by SPM instead of the standard MNI brain did not result in different clusters in any analyses.

#### Functional analyses

fMRI data were analyzed using hierarchical random-effects models as implemented in SPM. At the subject-specific first level, fMRI time series were regressed onto a general linear model (GLM) containing regressors which represented the time periods of the feedback task (Figure 1B): cue (1 s), imagination phase separately for self and other (4 s), rating phase (4 s), feedback phase separately for self and other (2 s), and two motor regressors for button presses with the left and the right hands (0 s). This resulted in 8 regressors for each of the four scanning runs. The imagination phase regressors for self and other were parametrically modulated by the respective first own ratings. The feedback phase regressors for self and other were modulated by the respective feedback ratings and the respective feedback discrepancies, to investigate the reward- and comparison-related feedback components, respectively (see Parametric modulation analyses). The model included trials with feedback discrepancies of zero. Trials in which participants failed to respond in time were not explicitly modeled because their number was negligible (see Table 2). The six motion correction parameters estimated from the realignment procedure were entered as covariates of no interest. Regressors were convolved with the canonical HRF and low frequency drifts were excluded using a high-pass filter with a 128 s cutoff. At the group level, we performed separate flexible factorial designs for the following conditions: feedback phase, imagination phase as well as parametric modulators for feedback ratings and feedback discrepancies. Specifically, we used flexible factorial designs including the following factors: a subject-specific constant, a group factor (culture: German/Chinese), and the interaction of group and condition (feedback target: self/other). All coordinates are reported in MNI space and activations are displayed on the standard MNI reference brain.

#### Parametric modulation analyses

We investigated trial-by-trial fluctuations in brain activity during the feedback phase, which correlated with two different components of social feedback: reward- and comparison-related components. We split trials according to feedback target (self/other) for each participant.

To detect activity related to social comparison we used parametric modulators of feedback discrepancies. We used the full parametric range of feedback ratings and feedback discrepancies across all trials (i.e., across trials with desirable and undesirable feedback and trials with feedback discrepancies of zero).

Activity related to the reward-related component of social feedback was operationalized as activity that correlated positively with feedback ratings for self. This approach follows a previous operationalization of reward in relation to social feedback (Izuma et al., 2008). The rationale is that higher feedback rating (e.g., a feedback rating of 8.0 on “polite”) is more rewarding than a lower feedback rating (e.g., a feedback rating of 7.0). Note that feedback ratings for negative traits were reverse-coded. That is, a high feedback rating indicated high self-relevant social reward (i.e., feedback that a positive trait applied to the self or that a negative trait did not apply to the self) and a low feedback rating indicated low self-relevant social reward. Reward-related activity was conceptualized as being specific for the self. To rule out possible activity that merely relates to higher numbers or other unspecific effects, we subtracted activity that correlated with the feedback ratings for other (i.e., we searched for activity that showed a higher positive correlation with feedback ratings for self than with feedback ratings for other; this analysis is conceptually similar to the interaction analysis performed by Izuma et al., 2008).

Activity associated with the social comparison-related component of social feedback should correlate positively with feedback discrepancies, which were defined as the absolute differences between first own ratings and feedback ratings; i.e., feedback discrepancies captured how close feedback ratings were to participants' own ratings, regardless of the direction of the differences.

All regressors and modulators were entered independently into the design matrix, i.e., without the serial orthogonalization used as default in SPM (for a similar approach see Gläscher et al., 2010; Wunderlich et al., 2011). This ensured that only the additional variance that could not be explained by any other regressor was assigned to the respective effect and thus prevented spurious confounds between regressors.

#### Follow-up analyses

We performed follow-up functional region of interest (ROI) analyses to visualize interactions and to visualize correlations between neural activity and the parametric modulators (i.e., the betas of the parametric modulators for feedback ratings and the betas of the parametric modulators for feedback discrepancies). We used the marsbar toolbox for SPM (marsbar.sourceforge.net/) to extract parameter estimates within functional ROIs.

## Results

### Characteristics of participants

Groups did not differ with respect to sex, education, and time living without parents (see Table 1 for overview). Chinese and Germans did not differ with regard to age (all *p* > 0.05). However, participants in Berlin were older than participants in Beijing (Mann–Whitney *U* = 840.5, *p* = 0.001). We accounted for this difference in age by testing whether including age as a covariate had an influence on the ANOVA results. This was not the case. For simplicity we therefore report all analyses without age as covariate. The German participants in Beijing and the Chinese participants in Berlin did not differ in how long they had lived abroad and in how long they had learned the respective foreign language. When tested, the Chinese participants in Berlin had been sojourners in Germany for less than 2 years. All but one of the German participants in Beijing had been sojourners in China for less than 2 years (excluding the participant who had been in China for 4.5 years did not alter the results).

### Behavior

#### Updating behavior

**Figure 2:**
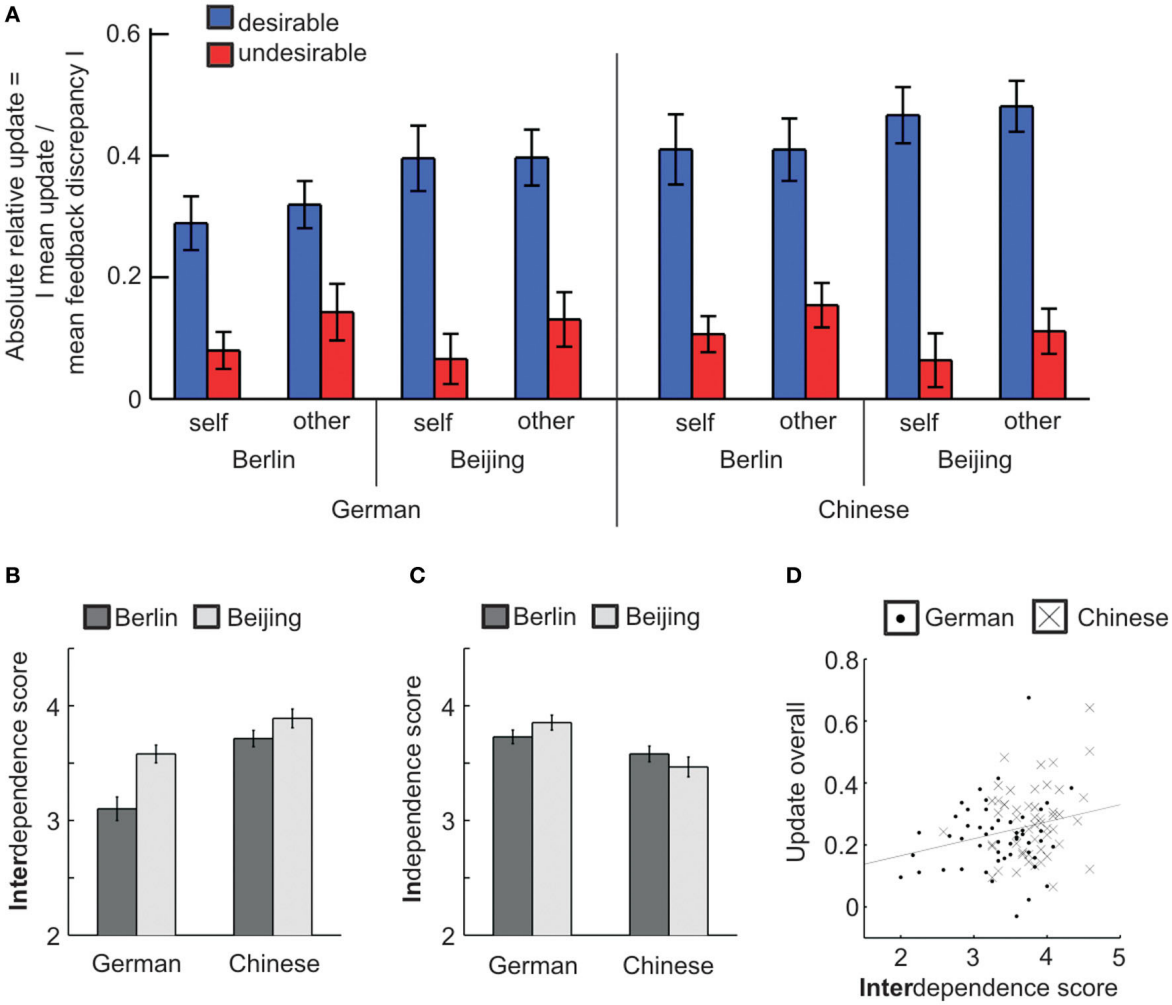
**Behavioral results—cultural difference in overall updating and cultural similarity in positively biased updating. (A)** Overall Chinese showed greater updating than Germans. Positively biased updating was evident across all participants, i.e., updates were higher for desirable compared with undesirable feedback. Additionally, relative absolute mean updates were higher for self- than for other-related feedback. **(B)** Chinese scored higher on interdependence than Germans. Participants living in Beijing scored higher on interdependence than participants in Berlin. **(C)** Germans scored higher on independence than Chinese. **(D)** Interdependence correlated with overall updates across all participants. Error bars refer to standard errors of the mean. See Table 2 for further details.

In our behavioral data we tested two main hypotheses: First, we tested whether Chinese took social feedback more into account than Germans by comparing relative absolute mean updates. Second, we tested whether participants of both cultural groups showed positively biased processing of social feedback—operationalized as greater relative absolute mean updates toward desirable compared with undesirable feedback. We found evidence supporting both hypotheses by comparing relative absolute mean updates in a 2 (target: self/other) by 2 (desirability: desirable/undesirable) by 2 (culture: German/Chinese) by 2 (place: Berlin/Beijing) ANOVA (Figure 2A and Table 2). In support of our first hypothesis, we found a significant main effect of culture with Chinese showing higher updates than Germans [*F*_(1, 100)_ = 4.64, *p* = 0.034, η^2^_*p*_ = 0.04]. In support of our second hypothesis, we found a significant main effect of feedback desirability with higher updates following desirable compared with undesirable feedback [*F*_(1, 100)_ = 107.2, *p* < 0.001, η^2^_*p*_ = 0.52]. We acknowledge that the effect size of the within-subject test for positively biased updating was larger than the effect size of the between-subject test for cultural differences in updating. Additionally, there was a significant main effect of feedback target with updates for other being higher than for self [*F*_(1, 100)_ = 4.07, *p* = 0.046, η^2^_*p*_ = 0.04]. No other main effects or interactions reached significance (all *p* > 0.05).

In addition, we tested whether the effect of culture was significant in the two groups that underwent fMRI (i.e., the two groups in Berlin). In a 2 (target: self/other) by 2 (desirability: desirable/undesirable) by 2 (culture: German/Chinese) ANOVA, the effect of culture reached significance albeit with a weak effect size [*F*_(1, 53)_ = 4.04, *p* = 0.049, η^2^_*p*_ = 0.07]. Furthermore, the main effect of desirability was significant [*F*_(1, 53)_ = 37.86, *p* < 0.001, η^2^_*p*_ = 0.42]. No other effects were significant (all *p* > 0.1).

For completeness, we also separately compared the updating behavior of the two groups that were tested in Beijing. The main effect of desirability was significant [*F*_(1, 47)_ = 87.68, *p* < 0.001, η^2^_*p*_ = 0.65] but the effect of culture failed to reach significance between these two smaller groups [*F*_(1, 47)_ = 0.37, *p* > 0.5, η^2^_*p*_ = 0.01]. No other effects reached significance. Since the overall ANOVA revealed no significant interaction of culture and place, we do not draw conclusions from this analysis but we acknowledge that future studies should test larger groups in China.

#### Individual differences and overall updating

To test whether updating correlated with individual variability in the endorsement of cultural values, we assessed participants' interdependence and independence scores (Singelis, 1994). The two scores did not correlate with each other (Pearson's *r* = −0.01, *p* > 0.9) and therefore we analyzed them separately. As expected, Germans were less interdependent and more independent than Chinese [2 (culture) by 2 (place) ANOVAs: interdependence: *F*_(1, 100)_ = 29.69, *p* < 0.001, η^2^_*p*_ = 0.23; Figure 2B; independence: *F*_(1, 100)_ = 14.44, *p* < 0.001, η^2^_*p*_ = 0.13; Figure 2C]. Participants in Beijing showed more interdependent self-construal compared with those in Berlin [*F*_(1, 100)_ = 14.90, *p* < 0.001, η^2^_*p*_ = 0.13]. The interaction of culture and place was at trend level for both scores [interdependence: *F*_(1, 100)_ = 3.20, *p* = 0.077, η^2^_*p*_ = 0.03; independence: *F*_(1, 100)_ = 2.88, *p* = 0.093, η^2^_*p*_ = 0.03].

In addition to trait measures on interdependence and independence, we also collected participants' score on the Rosenberg self-esteem scale (Rosenberg, 1965). Germans showed higher self-esteem than Chinese [*F*_(1, 100)_ = 5.24, *p* = 0.024, η^2^_*p*_ = 0.05]. Place had no effect on self-esteem (*p* > 0.1). Self-esteem scores correlated with independence [*r* = 0.32, *p* < 0.001, 95% confidence interval (CI) (0.14, 0.49)] but not interdependence scores [*r* = 0.02, *p* > 0.8; the two correlations differed significantly as assessed by Hotelling's *t*; *t*_(101)_ = 2.23; *p* = 0.028].

Interdependence scores correlated significantly with overall relative absolute mean updates [averaged across within-subject conditions; *r* = 0.25, *p* = 0.012, 95% CI (0.06, 0.42); Figure 2D]—more interdependent participants showed higher updating. The strength of this correlation did not differ between Germans and Chinese as assessed by Fisher's *z*-test (*p* > 0.7). Furthermore, the strength of the correlation between relative absolute mean updates and interdependence did not differ between updates for self and for other as assessed by Hotelling's *t* (*p* > 0.5). To test whether interdependence scores explained additional variance in updating beyond membership to the two cultural groups, we conducted a hierarchical regression on overall relative absolute mean updates including culture and interdependence scores as predictors. Interdependence scores explained additional variance at trend level [*F*_change(1, 101)_ = 3.06, *p* = 0.083]. The correlations between updating and independence and between updating and self-esteem were not significant (*p* > 0.5). The relationship between interdependence and updating remained significant when accounting for independence and self-esteem in a hierarchical regression [*F*_change(1, 100)_ = 6.46, *p* = 0.013].

Taken together, participants with more interdependent self-construal took social feedback more strongly into account. Participants in our sample and task showed positively biased feedback processing regardless of cultural group. The place where participants were tested had no effect on updating behavior.

#### Additional behavioral results—direction of updates, trait valence, first ratings, feedback discrepancies, perceived similarity scores, and memory errors

As expected, participants changed their ratings on average toward the feedback; they increased their ratings for desirable feedback and decreased their ratings for undesirable feedback as indicated by positive and negative relative mean updates, respectively [mean relative updates: self-desirable = 0.39, *SD* = 0.26; one-sample *t*-test against zero *t*_(103)_ = 15.0, *p* < 0.001; self-undesirable = −0.08, *SD* = 0.18; *t*_(103)_ = −4.4, *p* < 0.001; other-desirable = 0.40, *SD* = 0.23; *t*_(103)_ = 17.5, *p* < 0.001; other-undesirable = −0.14, *SD* = 0.21; *t*_(103)_ = −6.6, *p* < 0.001; see Table 2 for relative absolute mean updates separated according to group membership].

We explored whether the valence of the trait adjectives had an effect on updating. We split update scores according to the valence of the trait words and included valence as an additional factor in the ANOVA [resulting in a 2 (trait valence: positive/negative) by 2 (target: self/other) by 2 (desirability: desirable/undesirable) by 2 (culture: German/Chinese) by 2 (place: Berlin/Beijing) ANOVA on relative absolute mean updates]. The main effects of desirability and of culture were still significant [desirability: *F*_(1, 99)_ = 65.70, *p* < 0.001, η^2^_*p*_ = 0.40; culture: *F*_(1, 99)_ = 4.67, *p* = 0.03, η^2^_*p*_ = 0.05]. Additionally, there was a significant main effect of valence [*F*_(1, 99)_ = 16.66, *p* < 0.001, η^2^_*p*_ = 0.14] with updates for negative trait words being higher than for positive trait words. There were no further significant effects (all *p* > 0.05). Thus, although there was a significant main effect of valence, valence did not significantly interact with any other factor.

We tested for differences in participants' first self- vs. other-ratings in a 2 (feedback target: self/other) by 2 (culture: German/Chinese) by 2 (place: Berlin/Beijing) ANOVA. We found a significant main effect of feedback target with self-ratings being higher than other ratings across all participants [*F*_(1, 100)_ = 7.57, *p* = 0.007, η^2^_*p*_ = 0.07; Supplementary Figure S1; see Table 2 for mean first ratings separated according to group membership]. There were no further significant main effects or interactions (all *p* > 0.1). Thus, we found evidence for a positivity bias toward the self in participants' initial ratings, which did not differ across culture or place in our sample.

We excluded that the observed effects of culture and desirability on relative absolute mean updates were due to differences in feedback discrepancies (which were manipulated according to a random number generator). In a 2 (target: self/other) by 2 (desirability: desirable/undesirable) by 2 (culture: German/Chinese) by 2 (place: Berlin/Beijing) ANOVA on mean feedback discrepancies, no factor involving culture reached significance (all *p* > 0.3), thus excluding that the cultural difference in updating was driven by feedback discrepancies. In the ANOVA on feedback discrepancies, there was a significant main effect of desirability [*F*_(1, 100)_ = 7.51, *p* < 0.01, η^2^_*p*_ = 0.07] and a significant main effect of target [*F*_(1, 100)_ = 5.75, *p* < 0.05, η^2^_*p*_ = 0.05]. These latter effects arose because participants showed higher first ratings for themselves than for the other person (see previous paragraph) and because the random determination of the feedback discrepancies was necessarily based on these first ratings (mean feedback discrepancies across all four groups: self-desirable = 1.81, *SD* = 0.45; self-undesirable = 2.02, *SD* = 0.38; other-desirable = 1.81, *SD* = 0.46; other-undesirable = 1.9, *SD* = 0.37). Importantly, these small differences in feedback discrepancies did not drive the observed main effect of desirability for updating since an ANOVA on pure absolute mean update scores (i.e., without normalization with respect to feedback discrepancies) revealed a highly significant effect of desirability [*F*_(1, 100)_ = 120.68, *p* < 0.001, η^2^_*p*_ = 0.55; pure absolute mean updates across all four groups: self-desirable = 0.67, *SD* = 0.44; self-undesirable = 0.17, *SD* = 0.36; other-desirable = 0.71, *SD* = 0.43; other-undesirable = 0.24, *SD* = 0.39]. However, we acknowledge that the small difference in feedback discrepancies between self and other may have influenced the reported main effect of relative updates being higher for self than for other, which was not part of our a priori hypotheses. [The main effect of target was at trend level in the ANOVA on pure absolute mean updates: *F*_(1, 100)_ = 3.58, *p* = 0.06, η^2^_*p*_ = 0.03]. Therefore, we do not further interpret the main effect of target on updating.

In addition to trait measures on interdependence, independence, and self-esteem, participants indicated how similar they perceived the other person on a Likert scale. A 2 (culture) by 2 (place) ANOVA on similarity ratings did not reveal significant effects (*p* > 0.1). We have previously shown for the German participants in Berlin (Korn et al., 2012) that first self-ratings correlated with self-esteem scores and that first other-ratings correlated with how similar participants perceived the other person. These correlations held across all participants [self-ratings and self-esteem: *r* = 0.52, *p* < 0.001, 95% CI (0.36, 0.65); other-ratings and perceived similarity: *r* = 0.46, *p* < 0.001, 95% CI (0.29, 0.60)].

In a memory test, participants recollected the feedback ratings they had received. Similar to updates, mean absolute memory errors were subjected to a 2 (target: self/other) by 2 (desirability: desirable/undesirable) by 2 (culture: German/Chinese) by 2 (place: Berlin/Beijing) ANOVA. As expected, we found a significant main effect of feedback target with memory errors being smaller for self- compared with other-related feedback [*F*_(1, 97)_ = 71.83, *p* < 0.001, η^2^_*p*_ = 0.43]. There was also a significant main effect of culture with memory errors being smaller for Germans compared with Chinese [*F*_(1, 97)_ = 4.27, *p* = 0.041, η^2^_*p*_ = 0.04]. No other effects reached significance (all *p* > 0.05). Thus, positively biased updating seemed to be unrelated to memory. The cultural difference in memory is unlikely to have influenced cultural differences in updating for three reasons. First, memory performance did not correlate with updating behavior (*p* > 0.6). Second, memory performance did not correlate with interdependence or independence scores (*p* > 0.2). Third, the group with better memory performance should theoretically show higher updating. But Germans, who had better memory, showed smaller updating.

### fMRI

#### Cultural difference in neural feedback processing

Based on previous findings, which showed that culture influences ACC/MPFC activity during trait judgments (Zhu et al., 2007; Chiao et al., 2009a,b; Ng et al., 2010; Ma et al., 2014), we expected cultural differences in ACC/MPFC activity when participants received social feedback on personality traits.

**Figure 3:**
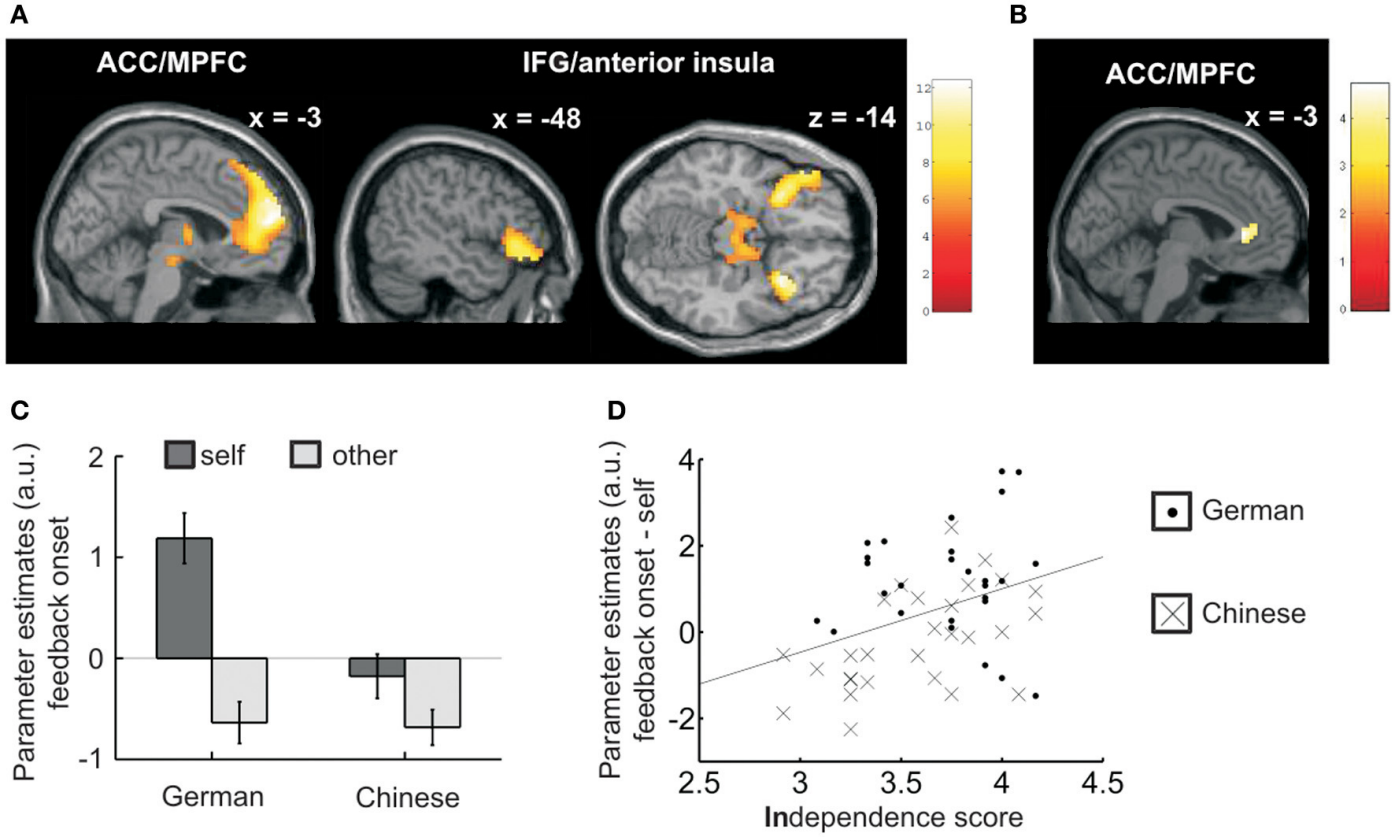
**fMRI results—cultural differences in BOLD signals when receiving feedback. (A)** When participants received social feedback, ACC/MPFC and IFG/anterior insula showed a main effect for feedback target (self > other) across all participants (*p* < 0.05 FWE corrected, cluster size > 15; Table 3). **(B)** When participants received social feedback, activity in the ACC/MPFC differed between Germans and Chinese. There was a culture (German/Chinese) by feedback target (self/other) interaction (*p* < 0.05 small volume corrected within the main effect shown in **(A)**; initial contrast threshold for interaction: *p* < 0.001, uncorrected). **(C)** To illustrate the interaction contrast depicted in **(B)** we extracted parameter estimates within the ACC/MPFC for self- and other-related feedback separately for Germans and Chinese. **(D)** Parameter estimates for self-related feedback onsets within the ACC/MPFC correlated with independence scores. There was a trend indicating that the strength of the correlation between independence and parameter estimates might be stronger for Chinese compared with Germans. Error bars refer to standard errors of the mean.

We contrasted the time periods when participants received self- vs. other-related feedback. In line with numerous studies on self-referential processing, we found changes in blood oxygen level-dependent (BOLD) signals in the medial prefrontal wall and bilateral inferior frontal gyrus (IFG) [*p* < 0.05 family-wise error (FWE) corrected, cluster size > 15; Figure 3A; see Table 3 for a full list of activations]. Consistent with our hypothesis, a cluster in the ACC/MPFC showed a culture (German/Chinese) by feedback target (self/other) interaction {*p* < 0.05 small volume corrected within the main effect of self > other; initial threshold for interaction: *p* < 0.001, uncorrected; Figure 3B and Table 3; restricting the analysis of interaction effects to areas showing a main effect is a common procedure [see Deserno et al. (2012) for a recent example] and is valid since the two contrasts are orthogonal}. Parameter estimates of this ACC/MPFC cluster are plotted in Figure 3C to visualize the interaction. Note that deactivations with respect to the implicit baseline are commonly observed in self- and other-referential activity (Amodio and Frith, 2006) and we do not draw any conclusions from the fact that in Chinese parameter estimates for self-related activity were around baseline whereas for Germans they were positive. (For completeness, we also mention that for the interaction contrast no cluster survived whole brain correction. There were two clusters at a lowered threshold of voxel-wise *p* < 0.001, uncorrected, *p* < 0.1 FWE cluster correction: ACC/MPFC: –3, 32, 4; cluster size 63; hippocampus: –30, –25, –11; cluster size 61).

**Table 3:** **Significant activations in feedback onsets**.

	**Side**	**Peak voxel MNI coordinates (mm)**			**Cluster size (Voxel)**	**Peak t score**
		***x***	***y***	***z***		
**MAIN EFFECT: FEEDBACK ONSET: SELF > OTHER**						
IFG (orbital part)/anterior insula	R	33	17	−14	217	12.42
MPFC/ACC	L/R	−3	56	16	1198	12.19
IFG (orbital part)/anterior insula	L	−30	14	−17	437	11.48
Cerebellum	R	27	−82	−35	146	9.71
Cerebellum	L	−30	−82	−38	106	9.20
Midbrain	L/R	9	−10	−14	94	7.32
Thalamus	L/R	−3	−4	4	35	6.78
**MAIN EFFECT: FEEDBACK ONSET: OTHER > SELF**						
Precuneus/postcentral gyrus/superior temporal gyrus/supramarginal gyrus	L/R	9	−55	49	7946	10.48
Middle frontal gyrus—dorso-lateral PFC	L	−36	44	31	76	7.30
Middle frontal gyrus—dorso-lateral PFC	R	24	32	34	51	6.33
Precentral gyrus	L	−54	5	28	15	5.72
**INTERACTION: FEEDBACK ONSET: (SELF > OTHER) × (GERMAN > CHINESE)**						
MPFC/ACC	L/R	−3	32	4	44	4.58
**WHOLE-BRAIN CORRELATION WITH OVERALL RELATIVE ABSOLUTE MEAN UPDATES IN THE CONTRAST FEEDBACK ONSET: SELF > OTHER**						
TPJ	L	−42	−52	19	219	5.04
Cerebellum	L/R	−9	−40	−20	194	4.37

For main effects, clusters are whole-brain FWE-corrected for multiple comparisons at the voxel-level *p* < 0.05, cluster size > 15. For interaction effects, clusters are small volume corrected within main effect: feedback onset: self > other; initial threshold for interaction: *p* < 0.001, uncorrected. For the whole-brain correlation with overall relative absolute mean updates in the contrast feedback onset: self > other, clusters are FWE-corrected at the cluster-level *p* < 0.05; initial threshold for interaction: *p* < 0.001, uncorrected.

To relate activity ACC/MPFC in the region that showed cultural to individual variability in self-construal, we correlated parameter estimates for self-related feedback (from the functional ROI depicted in Figure 3B) with self-construal scores. We found a significant correlation with independence [*r* = 0.36, *p* = 0.007, 95% CI (0.10, 0.57); Figure 3D] but not with interdependence [*r* = −0.06, *p* > 0.6; the two correlations differed significantly as assessed by Hotelling's *t*; *t*_(52)_ = 2.30; *p* = 0.02]. Although parameter estimates for self-related feedback correlated with self-esteem [*r* = 0.33, *p* = 0.016, 95% CI (0.07, 0.54)], the relationship between parameter estimates and independence remained significant when accounting for self-esteem in a hierarchical regression [*F*_change(1, 52)_ = 4.58, *p* = 0.037]. At trend level, the strength of the correlation between independence and parameter estimates differed between Germans (*r* = 0.05, *p* > 0.7) and Chinese [*r* = 0.53, *p* = 0.004, 95% CI (0.20, 0.75); Fisher's *z* = 1.87; *p* = 0.061].

To explore the relationship between individual differences in updating behavior in the task (i.e., differences in overall relative absolute mean updates) and brain activity, we additionally conducted a whole brain covariate analysis in the contrast self- vs. other-related feedback. Activity in no region showed a positive correlation with overall updates but activity in the left temporo-parietal junction (TPJ) and bilateral cerebellum showed a negative correlation with updating behavior across both groups (Table 3; clusters are FWE-corrected at the cluster-level *p* < 0.05; initial threshold for interaction: *p* < 0.001, uncorrected.). The TPJ activity was in the vicinity of the TPJ region in which Ma et al. (2014) found a difference between East Asian and Western participants when they made judgments about social traits but not when they made judgments about non-social traits.

In sum, our results extend previous findings by showing that culture influences ACC/MPFC and TPJ activity during feedback processing.

#### Neural activity related to reward and social comparison

We searched for BOLD signals that correlated with reward- and comparison-related components in a trial-by-trial fashion. Activity associated with the reward-related component fulfilled two requirements: First, reward-related activity correlated positively with feedback ratings for self (i.e., higher feedback rating for self-indicated more rewarding social feedback). Second, reward-related activity was self-specific (i.e., we performed a contrast between the parametric modulators for feedback ratings for self and other).

**Figure 4:**
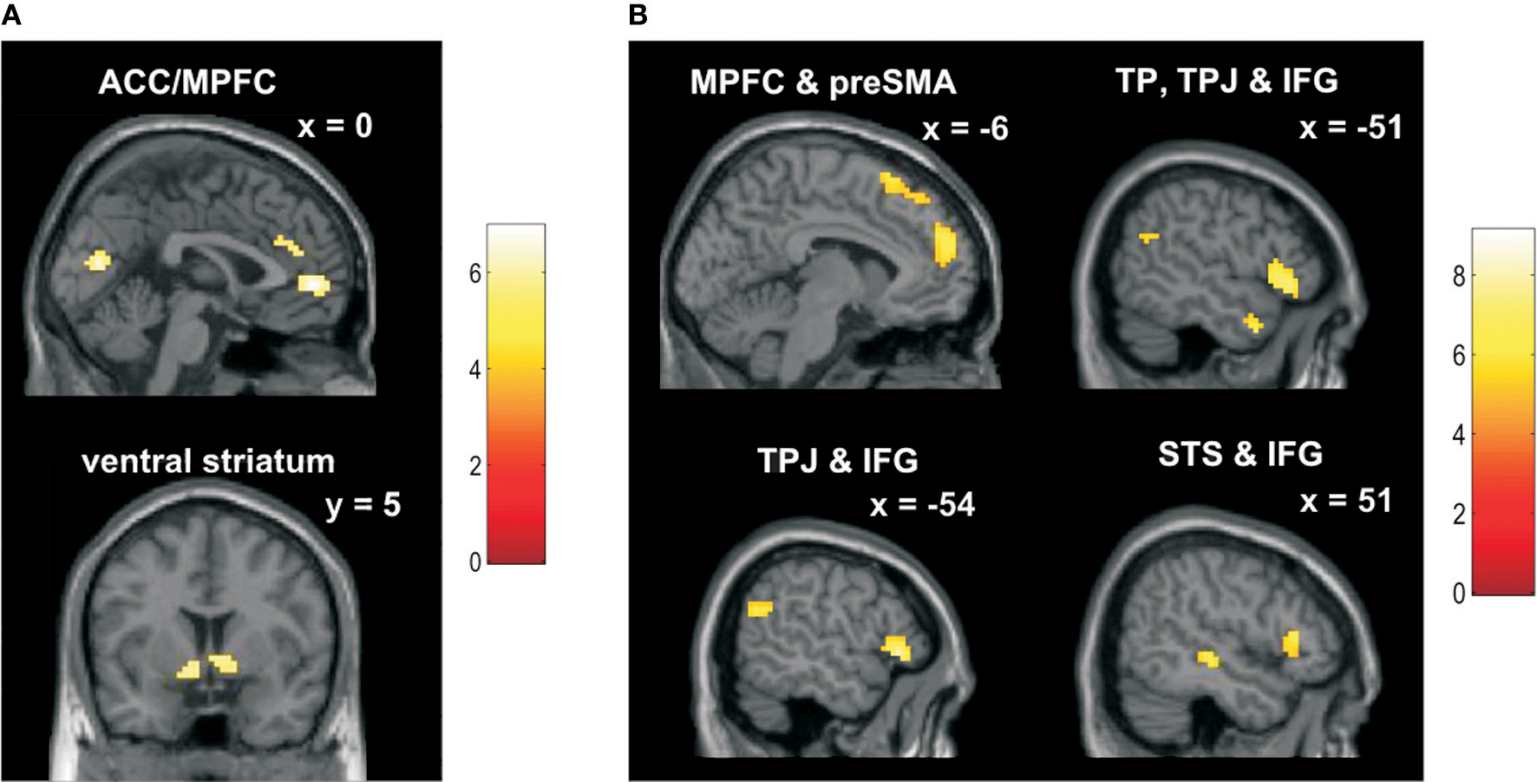
**fMRI results—BOLD signals of reward and comparison-related components of social feedback. (A)** Across all participants BOLD signal changes in ACC/MPFC and bilateral ventral striatum correlated with the reward-related component of feedback on a trial-by-trial basis at the time-point of feedback (*p* < 0.05 FWE corrected, cluster size > 15). See Table 4 for a full list of activations. Reward-related activity was identified in a contrast between the parametric modulators for feedback ratings for self and other. Thus, reward-related activity correlated positively with feedback ratings for self (e.g., a feedback rating of 8.0 on “polite” is more rewarding than a feedback rating of 7.0; feedback ratings for negative trait adjectives were reverse-coded). Reward-related activity was self-specific since it correlated more positively with feedback ratings for self than with those for other. **(B)** Across all participants BOLD signal changes in the following regions correlated with the comparison-related component of feedback on a trial-by-trial basis at the time-point of feedback: MPFC, preSMA/SMA, bilateral IFG (orbital part) extending into anterior insula, left TPJ, left TP and, right STS (*p* < 0.05 FWE corrected, cluster size > 15; Table 4). Comparison-related activity correlated positively with the feedback discrepancies for both self and other, i.e., with the absolute difference between participants' own views and the feedback they received.

Across all participants, the reward-related component was related to activity in the ACC/MPFC and bilateral ventral striatum; among other regions (*p* < 0.05 FWE corrected, cluster size > 15; Figure 4A and Table 4).

**Table 4:** **Changes in BOLD signal related to reward- and comparison-related components of social feedback**.

	**Side**	**Peak voxel MNI coordinates (mm)**			**Cluster size (Voxel)**	**Peak t score**
		***x***	***y***	***z***		
**FEEDBACK RATING (TRIAL-BY-TRIAL CORRELATION): SELF > OTHER**						
ACC/MPFC	L/R	0	50	1	43	6.69
Dorsal caudate	R	21	−19	16	62	6.78
Calcarine fissure	L/R	0	−76	13	98	6.48
Ventral striatum	R	6	11	−2	39	6.24
Cerebellum	L	−30	−73	−23	39	6.04
Ventral striatum	L	−6	5	−8	19	5.99
Precuneus	L	−12	−52	22	20	5.93
Lingual gyrus	L	−9	−58	1	19	5.84
ACC	L/R	0	41	19	31	5.79
Dorso-lateral PFC	L	−18	29	49	20	5.63
**FEEDBACK DISCREPANCIES (POSITIVE TRIAL-BY-TRIAL CORRELATION): SELF AND OTHER**						
MPFC	L/R	9	59	28	253	9.11
IFG (orbital part)/anterior insula	L	−54	26	−2	192	7.79
TP	L	−51	8	−32	24	7.04
Angular gyrus—TPJ	L	−60	−58	25	50	6.92
IFG (orbital part)	R	54	26	10	54	6.89
Anterior insula	R	33	20	−20	26	6.87
STS	R	51	−25	−5	32	6.87
Cerebellum	L	−27	−85	−35	16	6.14
preSMA/SMA	L/R	−6	20	64	83	6.13
**FEEDBACK DISCREPANCIES (NEGATIVE TRIAL-BY-TRIAL CORRELATION): SELF AND OTHER**						
Inferior parietal lobule	R	54	−37	52	114	8.12
Middle frontal gyrus	R	30	5	58	85	7.80
Inferior temporal gyrus	R	57	−49	−14	24	6.80
Inferior parietal lobule	L	−54	−40	43	142	6.67
IFG	L	−42	44	10	25	6.25
Middle occipital gyrus	L	−21	−61	40	18	5.73

Clusters are whole-brain FWE-corrected for multiple comparisons at the voxel-level p < 0.05, cluster size > 15.

Activity associated with the social comparison-related component was operationalized as activity that showed a positive trial-by-trial correlation with feedback discrepancies for self- and other-related feedback. That is, we searched for activity correlating with the absolute differences between participants' ratings and the feedback ratings they received (regardless of feedback desirability). We found comparison-related activity in MPFC, bilateral IFG extending into anterior insula, left temporal pole (TP), left TPJ, right superior temporal sulcus (STS), left cerebellum, and (pre-) supplementary motor area (preSMA/SMA; *p* < 0.05 FWE corrected, cluster size > 15; Figure 4B and Table 4).

#### Cultural comparisons of parametric modulators for reward- and comparison-related components

We explored differences between Germans and Chinese in neural activity associated with reward- and comparison-related components. No voxels were significant in any interaction contrast involving the factor culture (*p* < 0.05 FWE corrected, cluster size > 15). Furthermore, no clusters in any interaction contrast survived small volume correction within the relevant main effect (*p* < 0.05 small volume correction; initial contrast threshold for interaction: *p* < 0.001, uncorrected). Furthermore, we performed follow-up ROI analyses within the regions identified for reward- and comparison-related components by extracting parameter estimates for self- and other-related parametric modulators. These ROI analyses fulfilled to purposes: First, extracted parameter estimates illustrate the correlations of feedback ratings and feedback discrepancies with BOLD signals (Supplementary Figures S3A,B). Second, extracted parameter estimates were used to test for cultural differences in 2 (culture: German/Chinese) by 2 (feedback target: self/other) ANOVAs. We found no significant main effects or interactions involving the factor culture in any of the ROIs (all *p* > 0.1; *p*-values were adjusted using a Bonferroni correction for the number of ROIs; reward-related activity: 10 ROIs, comparison-related activity: 9 ROIs). In addition, we used a similar approach for extracted mean onset activity from the same ROIs but found no evidence for cultural differences (all *p* > 0.1).

In sum, the reward-related component of social feedback was related to the ACC/MPFC and ventral striatum. The comparison-related component (i.e., feedback discrepancies) correlated with activity in the MPFC, IFG, TPJ, STS, and TP. We did not find evidence for a cultural modulation of activity associated with reward- and comparison-related components in our sample and task.

#### Additional fMRI results—imagination phase

We focused our main fMRI analyses on the time period when participants received social feedback but our task also included a time period when participants made trait judgments of themselves and another person (imagination phase; Figure 1B). Since previous studies have mainly investigated cultural influences on ACC/MPFC activity when participants made trait judgments, we compared both time periods in a follow-up ROI-based approach.

We extracted parameter estimates during both time points within an ROI that was independently defined based on a recent study (Ma et al., 2014) comparing neural activity while Danish and Chinese participants made trait judgments of themselves and a public person (sphere with a radius of 15 mm centered at the MNI coordinate –4, 32, 0). We compared parameter estimates in a 2 (feedback target: self/other) by 2 (time period: feedback/imagination) by 2 (culture: German/Chinese) ANOVA. As expected self-related activity was higher than other-related activity [*F*_(1, 53)_ = 63.43, *p* < 0.001, η^2^_*p*_ = 0.55] and the factors culture and feedback target showed a significant interaction [*F*_(1, 53)_ = 10.97, *p* = 0.002, η^2^_*p*_ = 0.17; Supplementary Figure S2]. There was also a significant main effect of culture [*F*_(1, 53)_ = 5.26, *p* = 0.009, η^2^_*p*_ = 0.12], a significant time period by culture interaction [*F*_(1, 53)_ = 10.91, *p* = 0.002, η^2^_*p*_ = 0.17] as well as a significant three-way interaction [*F*_(1, 53)_ = 5.50, *p* = 0.023, η^2^_*p*_ = 0.09]. To qualify this three-way interaction we performed two separate 2 (self/other) by 2 (German/Chinese) ANOVAs for the feedback and imagination time periods. As expected from the analyses reported in the main text, the interaction of feedback target and culture was significant for the feedback time period [*F*_(1, 53)_ = 10.93, *p* = 0.002, η^2^_*p*_ = 0.17]. The same feedback target by culture interaction was also significant for the imagination time period [*F*_(1, 53)_ = 4.13, *p* = 0.047, η^2^_*p*_ = 0.07] but at to a lesser degree. Thus, the three-way interaction was qualified by a greater feedback target by culture interaction for the feedback phase compared with the imagination phase.

Taken together, in line with previous studies we found cultural influences on ACC/MPFC activity when participants made trait judgments. Our findings suggest that this cultural effect might be even stronger when participants receive social feedback.

#### Additional fMRI results—reward- and comparison-related activity

For completeness, we performed the reverse contrast to the contrast testing for reward-related activity, i.e., we searched for activity correlating with other-related feedback ratings more than with self-related feedback ratings. This contrast revealed no significant voxels (*p* < 0.05 FWE corrected, cluster size > 15). We also searched for regions correlating negatively with feedback discrepancies (see Table 4) and for activity correlating differentially for self- vs. other-related feedback discrepancies, i.e., self > other or other > self. These differential contrasts revealed no significant voxels (*p* < 0.05 FWE corrected, cluster size > 15).

For the German fMRI sample (Korn et al., 2012), we have previously shown in a conjunction analysis (i.e., in a test of the conjunction null hypothesis) that a region at the border of the MPFC and ACC was activated by both reward- and comparison-related components (*p* < 0.05 FWE corrected at cluster level, cluster-defining threshold of *p* < 0.0001). Using the same threshold, a conjunction for both Germans and Chinese revealed two clusters: a cluster at a similar location as shown before (MPFC/ACC: –3, 50, 10; cluster size 21) and a cluster in a more dorsal part of the ACC (3, 44, 25; cluster size 43). We note that at the more stringent threshold which we used to report clusters in the present study (*p* < 0.05 FWE corrected at voxel level, cluster size > 15), the conjunction revealed no regions of overlap.

In the previous study (Korn et al., 2012), we have shown that for the German fMRI sample the parameter estimates of the self-related absolute feedback discrepancies within the region revealed by the conjunction correlated with the behavioral update bias for self. In the MPFC/ACC region identified in the conjunction across both cultural groups, parameter estimates of the self-related absolute feedback discrepancies correlated with the update bias for self in Germans [Pearson's *r* = 0.42, *p* = 0.029, 95% CI (0.05, 0.70)] but not in Chinese (*r* = −0.02, *p* > 0.9). The difference in the strength of these correlations approached trend level (Fisher's *z* = 1.63; *p* = 0.103).

## Discussion

Cultural practices are shaped by social interactions but in most studies in cultural psychology or neuroscience participants perform tasks in the solitude of a test cubicle or fMRI scanner. Our design involves a face-to-face interaction of five peers of the same culture. By investigating how receiving feedback from these peers challenged participants' self-concept and their evaluation of others, we provide a novel approach to test for cultural differences in social conformity, positivity biases, and ACC/MPFC activity. For our behavioral analyses, we excluded confounds which might arise in the context of a real-life social interaction by testing both cultural groups in both countries.

On the behavioral level, we found that Chinese—compared with Germans—conformed more to social feedback on their own character traits and those of another person. Across both cultures more interdependent individuals showed higher conformity. However, cultural group membership did not influence positively biased feedback processing in our sample and task. Regardless of culture, participants changed their character trait ratings of themselves and of one of their interaction partners more toward desirable than toward undesirable feedback.

On the neural level, MPFC activity differed between Germans and Chinese and correlated with independence scores, when participants received social feedback. Additionally, exploratory analysis revealed that activity in the left TPJ correlated negatively with overall updating scores. As in our previous report of the German fMRI subsample (Korn et al., 2012), we found a tight link between the relevant task variables and neural activity. The reward-related component correlated with activity in regions previously implicated in social and non-social reward processing (i.e., ACC/MPFC and ventral striatum) (Fehr and Camerer, 2007; Izuma et al., 2008; Beckmann et al., 2009). The comparison-related component correlated with activity in the mentalizing network (i.e., MPFC, TPJ, STS, IFG, and TP) (Mar, 2011; Frith and Frith, 2012). Within our sample, we did not find evidence for a cultural influence on neural acitivity related to the reward- and comparison-related components.

### Cultural influences on social conformity

Western culture emphasizes that individuals should view their own character traits independently from the opinion of others. In contrast, East Asian culture emphasizes that individuals are interconnected. This difference in cultural values has been used to explain why members of interdependent cultures show higher conformity when receiving social information about the lengths of two lines (Bond and Smith, 1996). Unlike objective physical properties, character traits are open to interpretation and directly relevant for the assessment of the self or of others. Our results relate conformity about social feedback on character traits to differences in interdependence—both on the cultural and on the individual level. Our finding that Chinese changed their trait ratings more than Germans also fits well with the observation that East Asians perceive character traits as more malleable than Westerners (Choi et al., 1999) and with previous research on cultural differences in consensus motives (Fu et al., 2007). In our study, social feedback was provided by peers. Previous research indicates cultural differences in the relation to social hierarchies (Freeman et al., 2009; Lieuw et al., 2011). Based on this research, we would expect that receiving social feedback from a social superior would lead to a larger cultural difference in social conformity (i.e., East Asians would show considerably more social conformity than in the current task whereas Westerners would show rather similar levels of conformity).

In addition to culture, insecurity and information from an in-group (vs. an out-group) can lead to higher conformity (Bond and Smith, 1996). Living in a foreign culture might trigger a general state of insecurity and meeting compatriots in a foreign country might create strong in-group feelings (Sam and Berry, 2010; Heine, 2012). Furthermore, individuals who move abroad tend to be more independent than those who stay in their home country (Kitayama et al., 2006, in press) and may thus not be completely representative of their culture. For these reasons, we obtained behavioral data from both groups in both countries and could directly test for possible influences of the place where participants were tested. Our data did not provide any support that place modulated social conformity.

### Absence of cultural influences on positivity biases

Our results on social feedback processing provide a novel approach to the extensive debate on whether East Asians do or do not show similar degrees of positivity biases as Westerners (Sedikides et al., 2003, 2007; Heine et al., 2007; Heine and Hamamura, 2007). One of the main arguments centers on how positivity biases, and self-enhancement in particular, should be measured. Many studies used trait measures (e.g., the Rosenberg self-esteem scale) or compared how participants evaluated themselves and an “imagined” person from a reference group (Heine and Hamamura, 2007). In a few studies, participants received feedback on their performance in a task (e.g., a creativity test) (Heine et al., 2001). Success or failure feedback was then related to persistence on the task. Here, we conceptualized a positivity bias in terms of larger changes in character trait ratings toward desirable vs. undesirable feedback. This operationalization confers the following advantages: First, since we analyzed how ratings change and not ratings *per se*, we reduced possible confounds arising from cultural differences in completing Likert scales (Heine, 2012). Second, participants received feedback from persons with whom they had face-to-face contact and did not have to compare themselves to an “imagined” other person (i.e., reference-group effects were excluded) (Heine, 2012). Third, our approach combines two aspects of previous studies on positivity biases: character evaluations and processing of positive vs. negative feedback.

On the other hand, the finding that participants in our study showed positively biased updating when receiving feedback for themselves and for another person (i.e., one of the peers from the social interaction) may indicate differences with respect to previously reported positivity biases. While not all treatments of positivity biases involve self-other-comparisons, many definitions of positivity biases are based on a direct or indirect comparison of the self to an (often average) other person [see Beer and Hughes (2009) and Sui et al. (2009) for two neuroscience examples and Heine and Hamamura (2007) for an overview of the behavioral literature]. For example, the self-advantage in face recognition relies on a self-other-comparison (i.e., faster reaction times to one's own compared to a familiar face). In this context, we want to stress that our finding of higher initial self- vs. other-ratings replicate previous findings of indirect self-enhancement. But in the absence of differential updating for self and other, evidence is insufficient to label the observed updating pattern “self-enhancing.” Often a positivity bias for the self can extend to close others (but not to non-close others) (Hughes and Beer, 2012). Possibly, such a spread was facilitated in our design by the face-to-face interaction and by the fact that self- and other-related feedback was temporally intermixed. We expect that changing the relationship between the self and the other person (e.g., by using an in-group/out-group manipulation) would provide evidence for differences in self- vs. other-related updating, which might in turn be modulated by culture.

We found positively biased updating across both cultural groups in our sample of Germans and Chinese. Furthermore, we found no cultural differences in participants' inclination to rate themselves higher than the other person. Our findings are in line with evidence showing that American and Chinese individuals sought similar degrees of self-enhancing and self-improving feedback (Gaertner et al., 2012). Similarly, both Westerners and East Asians show a self-advantage in the perceptual domain. They respond faster to their own face than to faces of a familiar other. Taken together with our results, this suggests that an initial processing advantage for self-related stimuli may be intimately related to a processing advantage for higher-level information that depicts the self (and others) in a desirable light.

Overall, in the debate on whether East Asians do or do not show similar degrees of positivity biases, our findings are more in support of a pancultural expression of positivtiy biases (Sedikides et al., 2003, 2007). Nevertheless, we replicate findings showing higher trait self-esteem in Westerners compared with East Asians (Heine and Hamamura, 2007). Future studies have to corroborate whether our findings extend to Americans and Japanese since studies reporting cultural differences in positivity biases have mainly compared these two cultures (Heine and Hamamura, 2007).

Our design can be easily adapted to probe various cultural influences on feedback processing. Since close others (e.g., family members, friends, or colleagues) are especially important for interdependent individuals (Markus and Kitayama, 2010), cultural differences in positively biased feedback processing might emerge when feedback is given by close others and not by unrelated peers as in the present study. Importantly, previous studies showed a strong modulation of cultural differences in relation to social hierarchies (Freeman et al., 2009; Ma and Han, 2009; Lieuw et al., 2011). Including pictures of a superior other (e.g., a participant's boss) in the assessment of the self-advantage in face perception reduces the reaction time advantage for participants' own faces for East Asians but not for Westerners (Ma and Han, 2009; Lieuw et al., 2011). Following from these results, we predict that in our task receiving feedback from a superior would reduce the positivity bias in East Asians but not in Westerners. In addition, since modesty has been related to cultural differences in self-cement (Cai et al., 2011), future studies should address whether modesty modulates positively biased updating.

### Cultural influences on ACC/MPFC activity

Our fMRI results extend previous findings of cultural differences in ACC/MPFC activity during trait judgments (Zhu et al., 2007; Chiao et al., 2009a,b; Ng et al., 2010; Ma et al., 2014). In East Asians—but not in Westerners—MPFC activity for trait-judgments about self and mother overlapped (Zhu et al., 2007; Wang et al., 2012). The same pattern has been replicated with bicultural individuals from Hong Kong who were primed with Chinese or Western cultural symbols (Ng et al., 2010). General vs. contextual trait judgments (e.g., “I am polite” vs. “I am polite when I talk to my mother”) activated the MPFC differently depending on participants' self-construal (Chiao et al., 2009a)—a result replicated by priming independence or interdependence in bicultural Asian Americans (Chiao et al., 2009b). Furthermore, a recent study has elegantly linked cultural differences in MPFC activity (at a region similar to the one found in the present study) to the greater sensitivity of East Asians to social comparison within an economic game (Kang et al., 2013). Importantly, in a recent study (Ma et al., 2014) with a similar sample size as ours, self-related MPFC activity was higher in Westerners than in East Asians. Since cultural differences are conceptualized as differences in social interactions between the self and others, ACC/MPFC activity should be especially prominent when individuals receive social information about the self, which is what we found. Germans showed higher self-related ACC/MPFC activity than Chinese during social feedback processing. Individual differences in independence correlated with ACC/MPFC activity. There was a trend which suggested that the strength of the correlation between independence and ACC/MPFC activity might be more pronounced in Chinese than in Germans. Future studies should investigate whether the observed correlation might be higher for East Asians in general or for individuals who live abroad. In addition to cultural differences in ACC/MPFC activity, the study by Ma et al. (2014) also found a cultural modulation of TPJ activity. TPJ activity was specifically linked to judgments about social traits but not to judgments about non-social traits. In our design, TPJ activity correlated negatively with individual differences in the amount of overall updating. Thus, both studies suggest a more general role of the ACC/MPFC (related overall to trait measures) and a more specific role of the TPJ (related to components of the tasks) for cultural influences on social cognition.

In addition to self-related activity, we explored cultural differences of the reward- and comparison-related components of social feedback processing. We replicated our previous results (Korn et al., 2012). Across all participants the reward-related component of social feedback correlated with activity in ACC/MPFC and ventral striatum; both of which are implicated in reward processing (Fehr and Camerer, 2007; Izuma et al., 2008; Beckmann et al., 2009). The social comparison-related component (i.e., feedback discrepancies) correlated with activity in MPFC, IFG, TPJ, STS, and TP; regions previously related to mentalizing (Mar, 2011; Frith and Frith, 2012).

Culture did not modulate activity associated with reward- and comparison-related components in our sample and task. Although discussions based on null results are necessarily limited, our present results suggest that members of both cultures might process these components similarly. Based on the behavioral effect of higher updating within the Chinese group, one could have expected cultural differences in neural activity associated with the comparison-related component. Possibly, the effect size of the behavioral difference in updating was not sufficiently large within the scanned groups to be detected in our fMRI data. Furthermore, our behavioral measure of conformity comprised two factors (self- vs. other-directed feedback as well as desirable vs. undesirable feedback), which may have limited our ability to detect a simple relationship between cultural differences in behavioral and neural expressions of conformity. Interestingly, updating behavior correlated with interdependence but independence correlated with ACC/MPFC activity and overall updating correlated with TPJ activity (We analyzed interdependence and independence scores separately since they did not correlate with each other across participants.) This pattern suggests that cultural differences on an individual level might be differentially expressed in commonly used trait measures, task-related behavior, and neural activity.

### Limitations and outlook

First, while the sample size of our study is similar other fMRI studies, it is rather small compared to many studies in cultural psychology. Additionally, we focused on only two cultures and investigated cultural differences mostly with respect to the prominent framework of self-construal (i.e., interdependence and independence). Importantly, culture cannot be simply equated with the notion of self-construal (Oyserman et al., 2002; Triandis and Suh, 2002), and concepts such as complexity, tightness, and hierarchy probably have important influences on social feedback processing. The real-life social interaction renders the assessment of a very large and diverse sample difficult. We propose that future studies could reduce the social interaction to confirm our results in a larger group and across more cultures, which differ in ways unrelated to self-construal.

Second, in order to ensure experimental control, participants received manipulated social feedback. Although no participant doubted the veracity of the feedback, participants used to overall quite desirable (or undesirable) social feedback might have experienced overall less desirable (or undesirable) feedback than usual. Therefore, future studies should consider using actual feedback to confirm our results.

Third, participants knew before they started to interact that they were going to receive social feedback from the other players because we wanted all participants to have the same expectations about the experiment. The knowledge about upcoming social feedback might have prompted participants to present themselves in a particularly favorable light, which in turn might have enhanced positively biased updating. It would be interesting to investigate whether unexpected social feedback leads to a reduction in positively biased updating.

Fourth, we acknowledge that obtaining fMRI data only from Chinese participants living in Berlin may have limited the ability to detect cultural differences. Thus, in accord with a previous study on Chinese living in the US, which did not find cultural modulation of self- and mother-related MPFC activity (Chen et al., 2013), our findings suggest that future studies should take a more dynamic approach and investigate longitudinal changes within individuals adapting to a foreign culture.

## Conclusions

Social interactions are highly complex and differ widely across cultures. The complexity of social interactions and the complexity of culture make it difficult to distill the theoretically relevant components. We have investigated social feedback processing in a real-life setting, which allowed us to separately quantify social conformity and a positivity bias in receiving desirable vs. undesirable feedback as well as neural activity in brain areas underlying social cognition. We argue that theoretical advances in cultural neuroscience require realistic tasks that nevertheless allow a quantitative decomposition of the task variables. Understanding the principles in which cultures differ may ultimately lead to improved cross-cultural contacts. Overall, in our study the commonalities between the two cultures were more striking than the differences. In sum, by relating social conformity, positivity biases, and self-related neural activity to the processing of social feedback obtained in a real-life interaction, we provide an essential step toward a unifying framework for understanding human culture.

### Conflict of interest statement

The authors declare that the research was conducted in the absence of any commercial or financial relationships that could be construed as a potential conflict of interest.
