# Supplementary material for: Cultural influences on social feedback processing of character traits
Source: Front Hum Neurosci. 2014 Apr 4;8:192. doi: 10.3389/fnhum.2014.00192 (PMC3983486; doi:10.3389/fnhum.2014.00192)
Supplement: Supplementary file 1 [file Presentation1.ZIP › supplementary table 1.pdf]

SUPPLEMENTARY TABLE 1. List of trait adjectives

German	Chinese	English
Positive trait adjectives		
aufrichtig	诚实的	honest
bescheiden	谦虚的	modest
diszipliniert	遵守纪律的	organized
effizient	有效率的	efficient
einfühlsam	敏锐的	empathetic
enthusiastisch	热心的	enthusiastic
fleißig	努力的	hard-working
freundlich	友善的	friendly
geistesgegenwärtig	沉着灵敏的	quick-witted
gelassen	轻松镇静的	composed
geschickt	老练的	skilled
gesellig	好交际的	sociable
großzügig	大方的	generous
hilfsbereit	乐于助人的	helpful
höflich	有礼貌的	polite
kompetent	有能力的	competent
kooperativ	愿意合作的	cooperative

kreativ	有创造力的	creative
lebenslustig	热爱生活的	fun-loving
locker	不慌不忙的	easy-going
loyal	忠实的	loyal
offen	坦率的	open-minded
ordentlich	整齐的	tidy
respektvoll	尊重人的	respectful
scharfsinnig	有洞察力的	astute
schlagfertig	反应敏捷的	articulate
selbstständig	独立的	self-reliant
sorgfältig	细心的	diligent
souverän	很有把握的	confident
spontan	自发的	spontaneous
tatkräftig	精力充沛的	dynamic
tolerant	宽容的	tolerant
vernünftig	理智 的	level-headed
verständnisvoll	充分理解的	understanding
vertrauenswürdig	可信任的	trustworthy
vielseitig	多才多艺的	versatile
weitsichtig	有远见的	perspicacious
wissbegierig	好学的	inquisitive

zielstrebig	有目标的	goal-oriented
zuverlässig	可靠的	reliable

Negative trait adjectives

aggressiv	好斗的	aggressive
ängstlich	胆怯的	anxious
arrogant	傲慢的	arrogant
bieder	呆板的	overly-conservative
chaotisch	乱七八糟的	chaotic
egoistisch	自私的	selfish
eitel	虚荣的	conceited
engstirnig	心胸狭窄的	narrow-minded
feige	胆小的	cowardly
gehässig	恶毒的	spiteful
großmäulig	爱吹牛的	loud-mouthed
heuchlerisch	虚伪的	two-faced
hinterhältig	奸猾的	conniving
humorlos	缺乏幽默感的	humorless
inkonsequent	前后不一致的	inconsistent
kalt	冷漠的	cold-hearted
launisch	喜怒无常的	moody

leichtsinnig	漫不经心的	foolhardy
nachtragend	怀恨在心的	unforgiving
naiv	天真的	naive
oberflächlich	肤浅的	superficial
opportunistisch	机会主义的	opportunistic
pedantisch	死板的	pedantic
rücksichtslos	毫无顾忌的	inconsiderate
scheu	害羞的	unassertive
stur	固执的	stubborn
träge	懒散的	lazy
unentschlossen	犹豫不决的	indecisive
ungeduldig	不耐烦的	impatient
unnahbar	不易亲近的	inapproachable
unpünktlich	不准时的	tardy
unsicher	缺乏自信的	insecure
unsympathisch	不讨人喜欢的	unpleasant
verschwenderisch	浪费的	wasteful
voreilig	仓促的	rash
voreingenommen	先入为主的	biased
wehleidig	自怜的	whiny
zickig	爱挑剔的	catty

zwanghaft	强迫的	obsessive
zynisch	冷嘲热讽的	cynical
Adjectives used during the training trials		
intelligent	聪明的	intelligent
unsportlich	不爱运动的	unathletic
