# Supplementary figures and images for: Cultural influences on social feedback processing of character traits
Source: Front Hum Neurosci. 2014 Apr 4;8:192. doi: 10.3389/fnhum.2014.00192 (PMC3983486; doi:10.3389/fnhum.2014.00192)

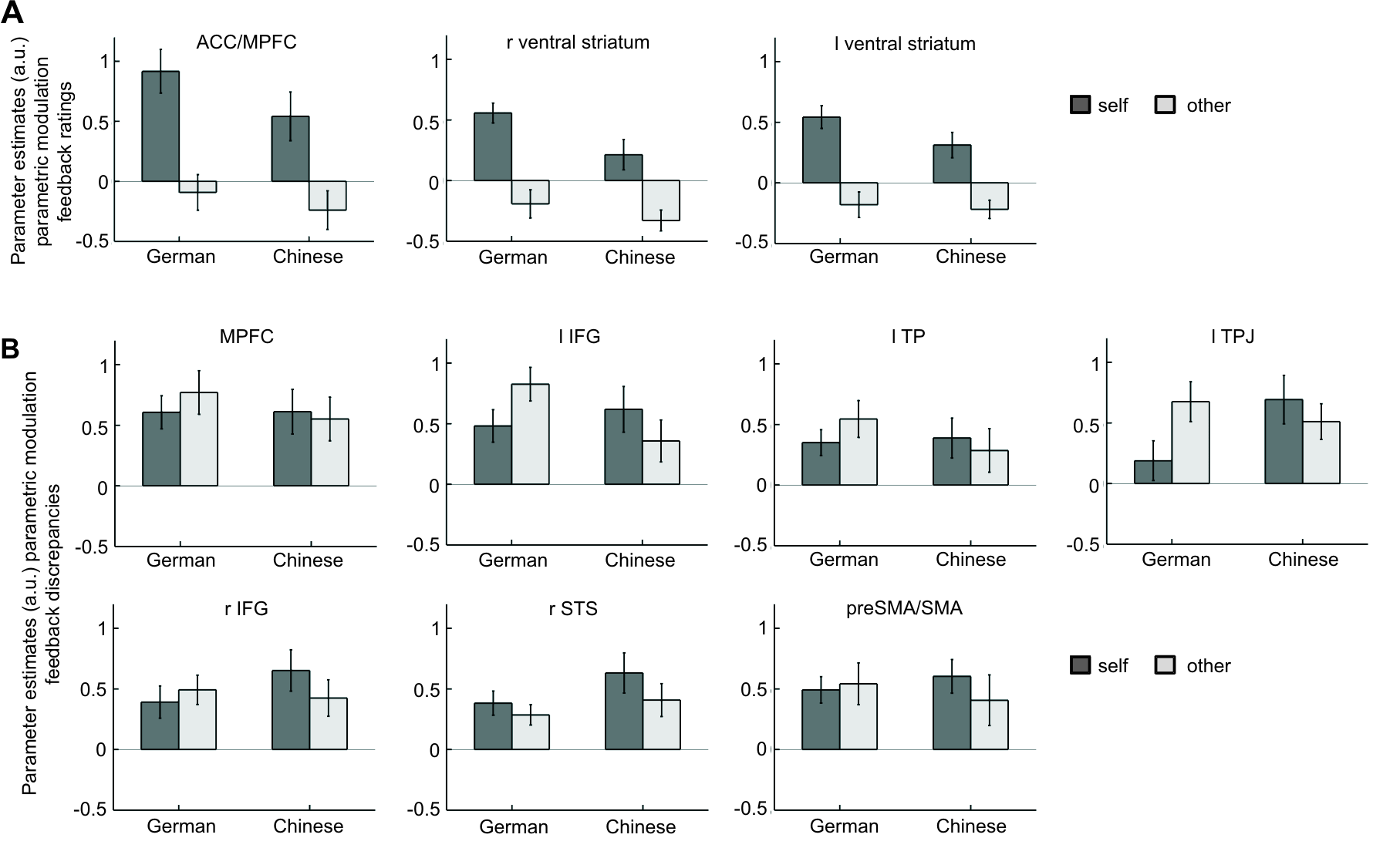

Supplement: Supplementary file 1 [file Presentation1.ZIP › supplementary figure 3.TIF]

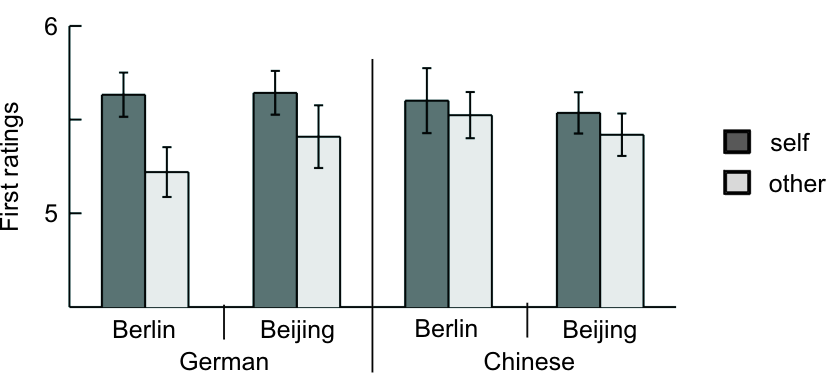

Supplement: Supplementary file 1 [file Presentation1.ZIP › supplementary figure 1.TIF]

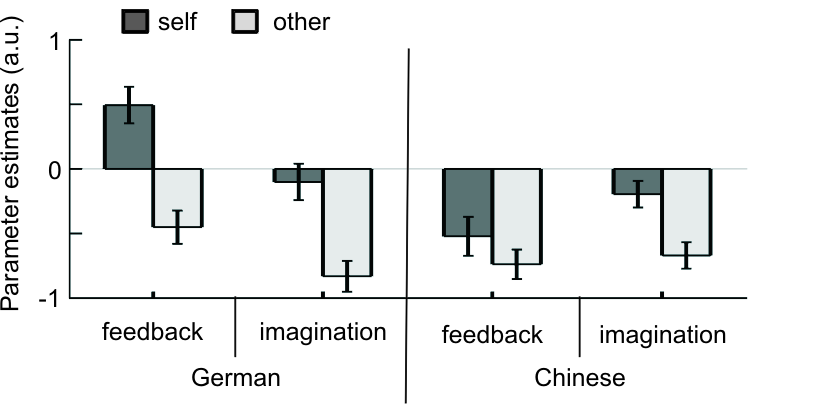

Supplement: Supplementary file 1 [file Presentation1.ZIP › supplementary figure 2.TIF]
